# Surface-enhanced Raman spectroscopy is capable of precise differentiation between re-dyed hair samples

**DOI:** 10.1038/s41598-023-34398-z

**Published:** 2023-05-01

**Authors:** Samantha Higgins, Dmitry Kurouski

**Affiliations:** 1 Department of Biochemistry and Biophysics, Texas A&M University, College Station, TX 77843 USA; 2 Department of Biomedical Engineering, Texas A&M University, College Station, TX 77843 USA

**Keywords:** Raman spectroscopy, Analytical biochemistry

## Abstract

Scalp hairs are readily present at most crime scenes because an average person sheds around 100 hairs a day. Forensic experts analyze hair found at crime scenes to identify suspects involved in a crime. Many people color their hair on a regular basis. Therefore, confirmatory analysis of hair colorants can be extremely useful in forensic investigation of hair evidence. However, most currently available methods for analysis of hair colorants are invasive, destructive, or not reliable. Surface enhanced Raman spectroscopy (SERS) is a minimally invasive, fast, and highly accurate technique that can be used to identify colorants present on hair. SERS is based on 106–108 enhancement of Raman scattering from molecules present in the close proximity to noble metal nanostructures. In this study, we investigate the extent to which SERS can be used to reveal coloration history of hair. We found that SERS enables nearly 100% identification of dyes of different color if those were applied on hair in the sequential order. The same accuracy was observed for colorants of different brand and type. Furthermore, SERS was capable of revealing the order in which two colorants were applied on hair. Finally, we demonstrated that SERS could be used to reveal hair coloration history if two randomly selected dyes of different color, brand and type were used to color the hair. These findings facilitate the need for forensic experts to account for hair that has been redyed and can be identified against a library of the same colorant combinations.

## Introduction

Hair evidence is readily available at a crime scene as many hairs are shed daily by the average person^1,2^. Hair shed from the scalp is the most commonly found type of hair evidence^3^. Scalp hair is often dyed for cosmetic purposes and may be used to disguise an identity. Also, hair colorants give criminals the opportunity to change their appearance.

The market for hair dye is projected to continue expanding to $36.2 billion by 2027^4^. As the market for hair dye grows so does the number of hair colorants available for public use. Due to this projected usage increase, there is a growing demand for forensic methods of hair analyses. Ideally such methods should be robust, reliable, minimally invasive, and non-destructive. At the same time, most of the current methods of analyses, such as high-performance liquid chromatography, gas chromatography, and mass spectrometry, damage hair specimens^5,6^. PCR analysis can be used to reveal the identity of a suspect through hair evidence. However, this approach requires soft tissue present on hair. It is also destructive to the evidence providing information only about the maternal relatives^7,8^. It has been proposed that a non-destructive method of UV–visible microspectrophotometry can be used for hair colorant analysis. However, this approach is highly laborious and can be used only after microscopic evaluation of hair^9^. Lednev group recently showed that Infrared spectroscopy could be used to differentiate between (i) colored and un-colored hair, (ii) different types (permanent *vs* semi-permanent) and (iii) brands of hair colorants^10^.

There is a growing body of evidence that surface-enhanced Raman Spectroscopy (SERS) can be used for a confirmatory and minimally destructive analysis of hair colorants^11,12^. SERS is based on 10^6^–10^8^ enhancement of Raman scattering from the colorants present on hair by noble metal nanostructures^13,14^. This technique has been broadly used in forensic science to analyse trace amounts of illicit drugs^15^, bodily samples^16^, and other trace elements^17,18^. Recently our group demonstrated that SERS could be used to distinguish more than 30 different colorants, as well as differentiate between different brands and types of colorants^19^. Finally, the researchers showed that SERS could be used for the automatic identification of hair colors.

Expanding upon this, we investigate the extent to which SERS can detect underlying dyes if the hair was re-colored afterwards. One can envision four different scenarios of hair coloration: (i) overlaying hair dyes with different colors, (ii) overlaying hair colorants of different brands, (iii) overlaying hair colorants of different types, and (iv) overlaying hair colorants that have different brand, type, and color. In the current study, we examine all these combinations using SERS couped to chemometrics to determine the accuracy with which SERS can be used to unravel coloration history of hair.

## Results and discussion

### SERS-based identification of overlying colorants with different color

**Figure 1:**
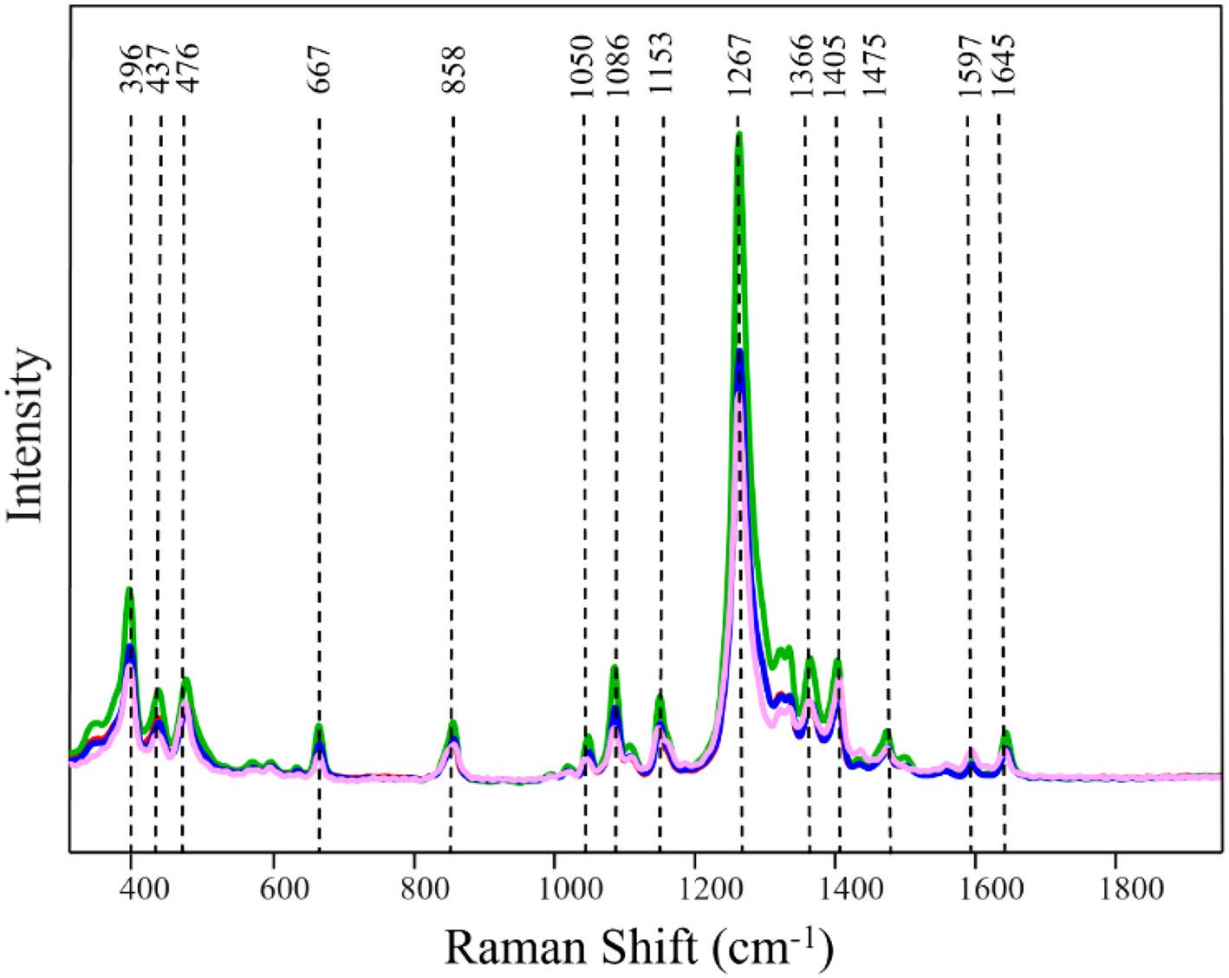
Normalized and baselined SERS spectra of single- and dual-dyes of different colors present on hair. Red trace (Wella semi-permanent blue under Wella semi-permanent purple), green trace (Wella semi-permanent purple under Wella semi-permanent blue), blue trace (Wella semi-permanent blue), pink trace (Wella semi-permanent purple).

We first examined the extent to which SERS can be used to differentiate the underlaying hair colorants of the same brand and type (semi-permanent or permanent) but different colors (blue and purple). For this, hair was first colored by Wella semi-permanent blue (WSBu) and re-colored by Wella semi-permanent purple (WSPu) (WSBuWSPu) that possessed very similar components to WSBu chemical composition. Next, we reversed the order of colorant application by first coloring hair with WSPu and then re-dying it with WSBu (WSPuWSBu). We acquired SERS spectra from all those four samples, Fig. 1. SERS spectra of WSBuWSPu, WSPuWSBu, WSPu and WSBu have distinct vibrational bands at 396, 437, 476, 667, 858, 1050, 1086, 1153, 1267, 1366, 1405, 1475, 1597, and 1645 cm^−1^. We observed only minor spectral differences that cannot be used for unambiguous differentiation between WSBuWSPu and WSPuWSBu, as well as between SERS spectra acquired from the hair colored with one and two dyes. To overcome this limitation, we used PLS-DA to investigate the accuracy of differentiation between all four classes of SERS spectra.

Our results show that WSBuWSPu, WSPu and WSBu can be identified with 100% accuracy, whereas WSPuWSBu can be predicted with 98% accuracy, Table 1. These results demonstrate that the application of two colorants creates a unique dye appearance on hair that is distinctly different from individual dyes used to color the hair (Figure S1). Our results also show that SERS can be used to identify the order of dyes application on hair, Table 2.

**Table 1:** Cross-validation matrix of SERS spectra acquired from hair with single and dual-dyes of different colors with TPR of SERS-based identification of hair colored with two dyes of different color.

	Actual class				
Predicted as	TPR, %	WSBuWSPu	WSPuWSBu	WSBu	WSPu
WSBuWSPu	100	50	1	0	0
WSPuWSBu	98	0	49	0	0
WSBu	100	0	0	50	0
WSPu	100	0	0	0	50

**Table 2:** Cross-validation matrix of two dyes of different color based on the order of their application on hair with TPR of SERS-based identification of the order of dye application on hair.

	Actual class		
Predicted as	TPR, %	WSBu under WSPu	WSPu under WSBu
WSBu under WSPu	100	50	0
WSPu under WSBu	100	0	50

### SERS-based identification of overlying colorants of different brands

**Figure 2:**
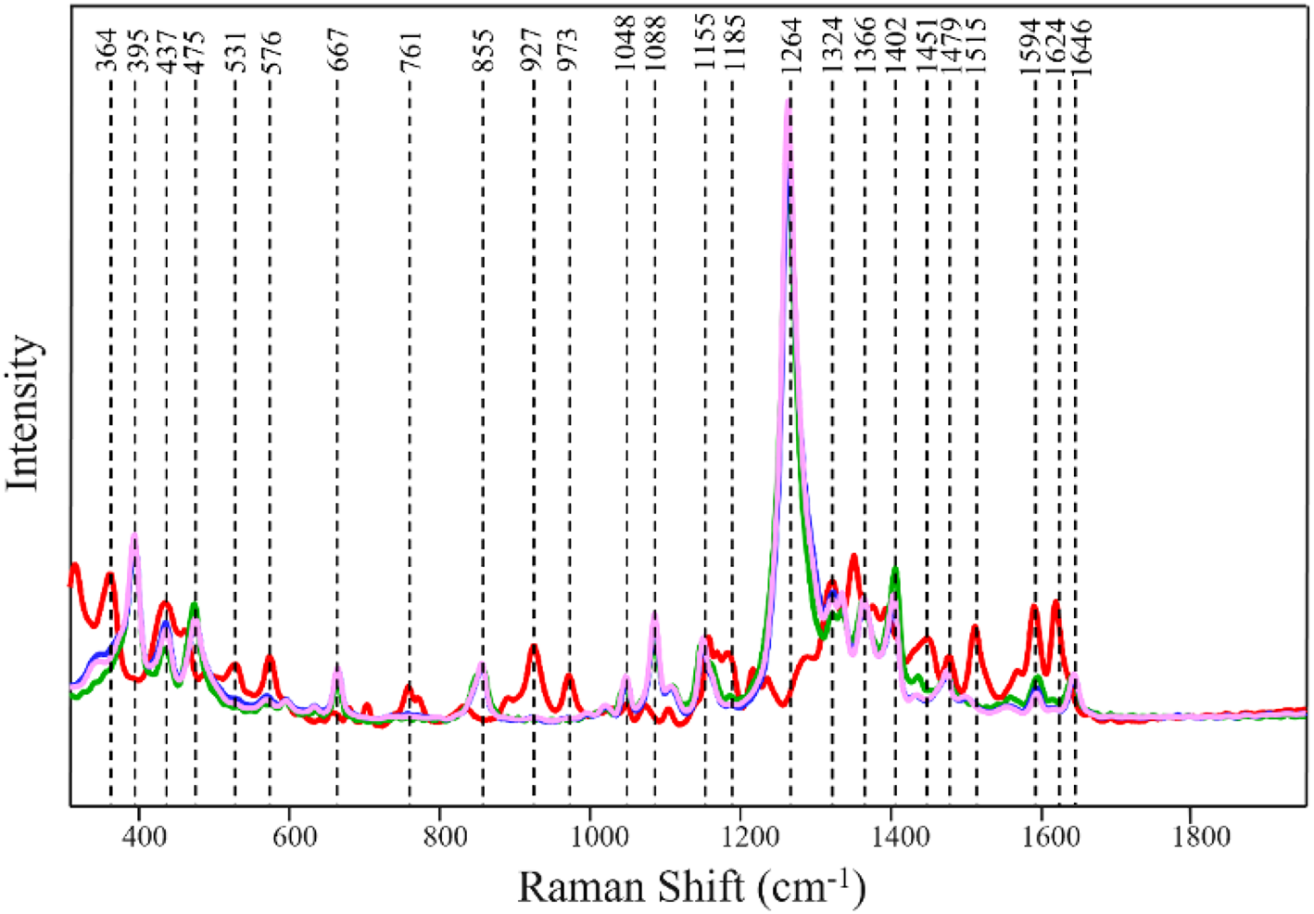
Normalized and baselined SERS spectra of single- and dual-colorants of different brands present on hair. Red trace (Ion semi-permanent purple), green trace (Wella semi-permanent purple), blue trace (Ion semi-permanent purple hair dye under Wella semi-permanent purple), pink trace (Wella semi-permanent purple hair dye under Ion semi-permanent purple hair dye).

We investigated the extent to which SERS can be used to differentiate the underlaying hair colorants of different brands. For this, we colored hair using Ion semi-permanent purple (ISPu) and then applied Wella semi-permanent purple (WSPu) on this hair (ISPuWSPu). We also reversed the order of dye application and first colored hair with WSPu and re-dyed this hair sample with ISPu (WSPuISPu). Next, we acquired SERS spectra from these hair samples, as well as from hair colored by WSPu and ISPu themselves, Fig. 2. SERS spectrum of ISPu has vibrational bands at 364, 437, 531, 576, 761, 927, 973, 1155, 1185, 1324, 1360, 1451, 1479, 1515, 1594, and 1624 cm^−1^, whereas SERS spectrum of WSPu exhibits distinct vibrational bands at 395, 475, 667, 855, 1048, 1088, 1264, 1366, 1402, and 1646 cm^−1^.

We found that both WSPuISPu and ISPuWSPu do not exhibit equally intense signatures of WSPu and ISPu. Instead, SERS spectra acquired from hair with two dyes dominate by the spectroscopic signatures of WSPu with no regards whether this colorant was under- or overlaying. These results demonstrate that an application of two colorants that belonged to different dye brands on hair creates the unique dye appearance that is distinctly different from individual colorants used to dye hair (Figure S2).

Utilization of PLS-DA enabled highly accurate identification of all four groups of SERS spectra, Tables 3 and 4. These results demonstrate that SERS can be used to identify coloration history of hair in regard to the brands of colorants used on hair. Our results also show that SERS can be used to identify the order of application of different brands on hair, Tables 3 and 4.

**Table 3:** Cross-validation matrix of SERS spectra acquired from hair with single and dual-dyes of different brands with TPR of SERS-based identification of hair colored with two dyes of different brands.

		Actual class			
Predicted as	TPR, %	ISPu	WSPu	ISPuWSPu	WSPuISPu
ISPu	100	50	0	0	0
WSPu	100	0	50	0	0
ISPuWSPu	100	0	0	50	0
WSPuISPu	100	0	0	0	50

**Table 4:** Cross-validation matrix of two dyes of different brand based on the order of their application order with TPR of SERS-based identification of the order of application of colorants of different brands on hair.

	Actual class		
Predicted as	TPR	ISPu under WSPu	WSPu under ISPu
ISPu under WSPu	100	50	0
WSPu under ISPu	100	0	50

### SERS-based identification of overlying colorants of different types

All hair colorants can be divided into two classes: permanent and semi-permanent. Permanent colorants are based on phenyldiamines that have different substituents around the aromatic ring. Their oxidation by hair developer causes formation of azo polymers, also known as Borowsky bases. These polymers develop strong covalent interactions with keratin on hair, which makes these colorants stay for a long time on hair. Semi-permanent colorants do not require hair developers. These colorants consist of one or several individual dyes that can be easily washed away from the hair.

**Figure 3:**
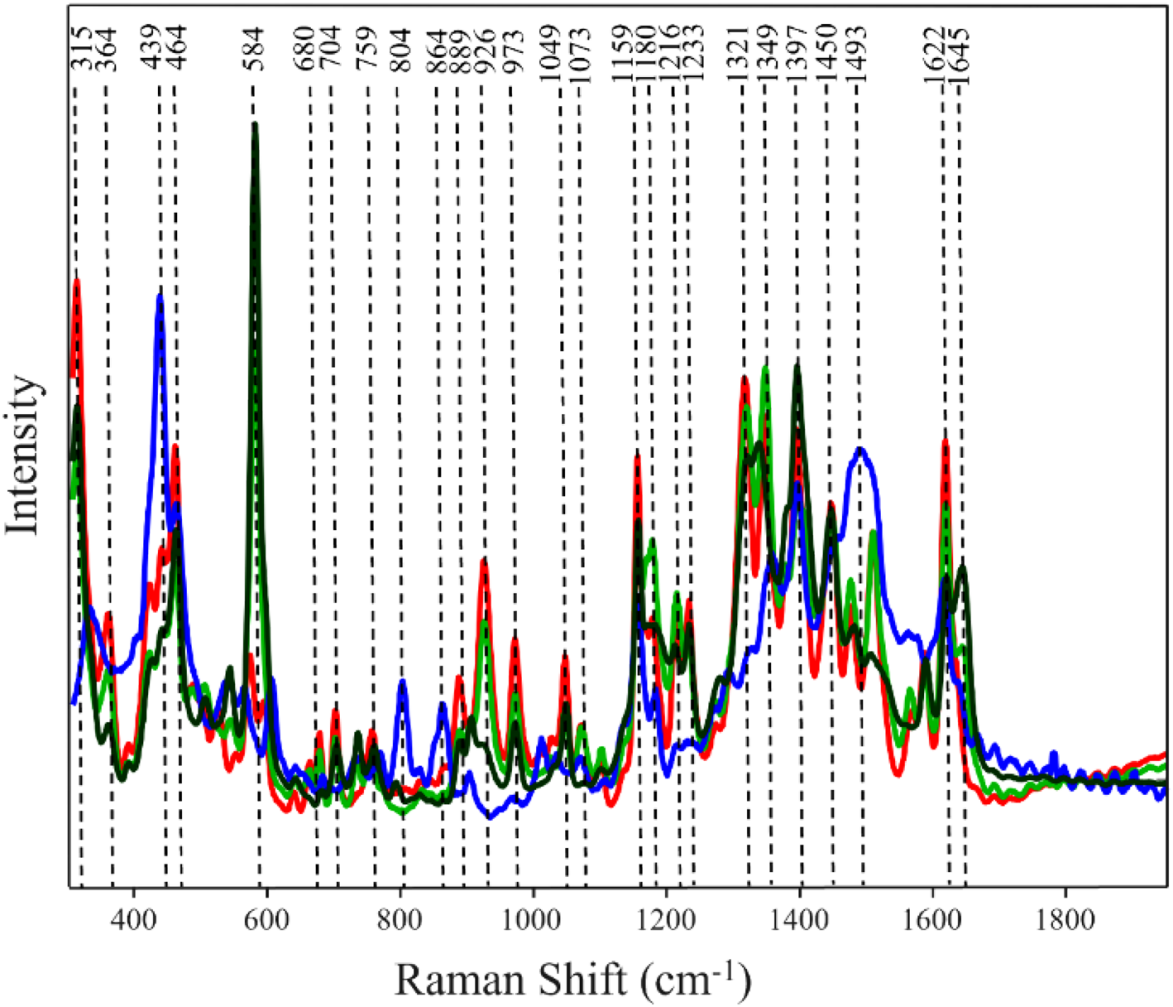
Normalized and baselined SERS spectra of single- and dual-colorants of different dye types present on hair. Red trace (Ion semi-permanent blue dye under Ion permanent blue dye), green trace (Ion permanent blue dye under Ion semi-permanent blue dye), blue trace (Ion permanent blue), black trace (Ion semi-permanent blue).

We first colored hair with Ion semi-permanent blue dye (ISBu) and then applied Ion permanent blue dye (IPBu) on the same hair (ISBuIPBu). We also reversed application of these colorants on hair (IPBuISBu), as well as dyed hair with ISBu and IPBu alone. SERS spectra acquired from these four hair samples are shown in the Fig. 3. SERS spectrum of IPBu exhibit intense bands at 439, 804, 864, and 1493 cm^−1^, whereas SERS spectrum of ISBu has vibrations at 464, 584, 704, 973, 1049, 1159, 1233 1397, 1450 and 1645 cm^−1^. SERS spectra of both ISBuIPBu and IPBuISBu exhibited higher intensities at 315, 464, 680, 704, 759, 889, 926, 973, 1159, 1321, 1349, 1622 cm^−1^, Fig. 3. Our results show that SERS spectra of both ISBuIPBu and IPBuISBu look very similar to ISBu with very little character of IPBu.

Utilization of PLS-DA enabled identification of all classes with 100% accuracy, Tables 6 and 7. These results demonstrate that SERS can be used to identify application of different types of colorants on hair, Tables 5 and 6, and Figure S3.

**Table 5:** Cross-validation matrix of SERS spectra acquired from hair with single and dual-dyes of different types of colorants with TPR of SERS-based identification of hair colored with two dyes of different types.

	Actual class				
Predicted as	TPR, %	ISBuIPBu	IPBuISBu	IPBu	ISBu
ISBuIPBu	100	50	0	0	0
IPBuISBu	100	0	50	0	0
IPBu	100	0	0	50	0
ISBu	100	0	0	0	50

**Table 6:** Cross-validation matrix of two dyes of different type based on the order of their application on hair with TPR of SERS-based identification of the order of application of colorants of different types on hair.

	Actual class		
Predicted as	TPR, %	IPBu under ISBu	ISBu under IPBu
IPBu under ISBu	100	50	0
ISBu under IPBu	100	0	50

### SERS-based differentiation of hair dyes of different color, brand, and type

**Figure 4:**
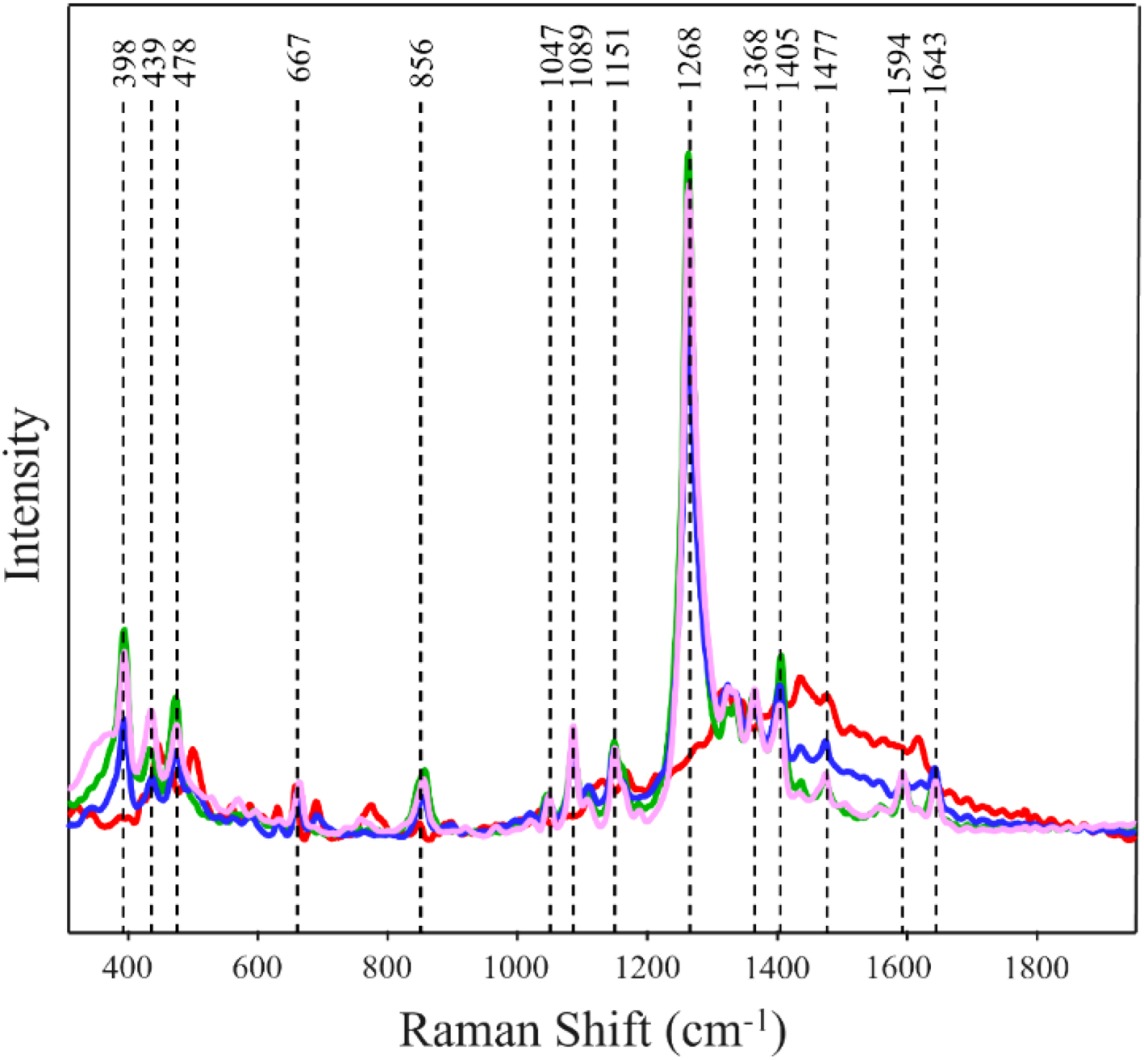
Normalized and baselined SERS spectra of single- and dual-colorants of different brand, type, and color. Red Trace (L’Oréal permanent red), green trace (Wella semi-permanent purple), blue trace (Wella semi-permanent purple hair dye under L’Oréal permanent red hair dye), pink trace (L’Oréal permanent red under Wella semi-permanent purple).

One may wonder whether SERS can be used to determine hair coloration history of two randomly selected dyes of different color, brand, and type. To answer this question, we first colored hair with Wella semi-permanent purple (WSPu) hair dye that was colored afterwards with L’Oréal permanent red (LPR) hair dye (WSPuLPR). We also altered the order of hair coloration by these two dyes and first colored hair with LPR then re-dying it afterwards with WSPu (LPRWSPu). We also colored hair with just LPR and WSPu. Next, we collected SERS spectra from WSPuLPR, LPRWSPu, LPR and WSPu, Fig. 4. We found that vibrational bands observed in the SERS spectrum of WSPuLPR and LPRWSPu primarily originated from WSPu with very little contribution of LPR (Figure S4).

PLS-DA was able to identify SERS spectra collected from all four classes with nearly 100% accuracy. The same accuracy was observed for the binary model built for WSPuLPR and LPRWSPu, Tables 7 and 8. These results demonstrate that SERS can be used to unravel hair dying history in regard to the color, brand and type of the colorants used to dye hair.

**Table 7:** Cross-validation matrix of SERS spectra acquired from hair with single and dual-dyes of different color, brand, and type of colorants with TPR of SERS-based identification of hair colored with two dyes of different color, brand, and type.

	Actual class				
Predicted as	TRP, %	LPR	WSPu	WSPuLPR	LPRWSPu
LPR	100	50	0	0	0
WSPu	100	0	45	0	0
WSPuLPR	100	0	0	50	0
LPRWSPu	100	0	5	0	50

**Table 8:** Cross-validation matrix of two dyes of different color, brand, and type based on the order of their application on hair with TPR of SERS-based identification of the order of application of colorants with different color, brand, and type.

	Actual class		
Predicted as	TPR, %	LPR under WSPu	WSPu under LPR
LPR under WSPu	100	50	0
WSPu under LPR	100	0	50

## Conclusion

Our results show that SERS is capable of unravelling coloration history of hair in regard to the dye color, brand, and type that was used to color hair. We also found that spectroscopic fingerprints of re-dyed hair largely represent one of the two dyes used to color hair. This can be explained by different Raman cross-section of colorants in such pairs of dyes. Thus, the colorant with larger Raman cross-section of dyes in it dominates in the SERS spectra acquired from hair with two colorants present on it. Therefore, application of chemometric analysis of spectra is required to reveal the information about the underlying hair colorant. One can expect that forensic application of the discussed above SERS-based approach will required a library of hair colorants with two and three individual colorants simultaneously present on hair to enable robust and reliable determination of hair coloration history.

## Materials and methods

### Hair coloring procedure

Blonde hair that was never colored prior to the experiments was collected from a hair salon in College Station, Texas from de-identified individuals. Hair was used as received without any pre-treatment or washing. It was cut in small ponytails of approximately the same density and tightened with elastics to minimize hair lost during dying and washing. Six total hair dyes were used to investigate the extent to which SERS could be used to determine the hair dying history, Table 9.

**Table 9:** Hair colorants used in this study.

Testing difference in:	Brand of hair dye	Commercial name	Color of hair dye	Type of hair dye
Color	Wella	Blue	Blue	Semi-permanent
Color	Wella	Wild Orchid	Purple	Semi-permanent
Brand	Ion	Radiant Orchid	Purple	Semi-permanent
Brand	Wella	Wild Orchid	Purple	Semi-permanent
Type	Ion	Sapphire	Blue	Semi-permanent
Type	Ion	Tanzanite	Blue	Permanent
Brand, type and color	Wella	Wild Orchid	Purple	Semi-permanent
Brand, type and color	L’Oréal	Chroma Ruby	Red	Permanent

All semi-permanent dyes were allowed to process approximately 45 min, and permanent hair dye was processed for ~ 60 min according to instructions provided by colorant manufacturers.

### Surfaced enhanced Raman spectroscopy

Each hair sample was coated with 5 µl of gold nanoparticles’ suspension (AuNPs). AuNPs were made in the laboratory according to the procedure developed by Esparza and co-workers^12^. These spherical nanoparticles had ~ 80 nm in diameter. Prior to utilization on hair, the suspension of AuNPs was centrifuged at ~ 5000 g for 10 min to concentrate AuNPs. Next, the pellet of AuNPs was re-suspended in DI water to remove detergent used for the nanoparticle synthesis. SERS spectra were acquired on a TE-2000U Nikon inverted confocal microscope equipped with a 20 × Nikon objective. The objective was used to focus the laser light (λ = 785 nm) generated by continuous wavelength laser on the sample. The same objective was used to collect scattered photons that were directed to a 50/50 light beam splitter and then passed to IsoPlane-320 spectrograph (Princeton Instruments) equipped with a 600 groove/mm grating. A long-pass filter (Semrock, LP-785RS-25) was used to cut off inelastically scattered photons. Laser power at the sample was ~ 1.8 mW. Spectral acquisition times were varying dependent on sample, but all were under 60 s. All reported SERS spectra were normalized and baselined. Spectral resolution was 2 cm^−1^.

### Data analysis

We used Matlab equipped with Partial Least Squares Differentiative Analysis toolbox (Eigenvector Research Inc) for statistical analyses of the collected SERS spectra. All spectra were pre-processed by baselining using a second order automatic weighted least squares, taking the first derivative of spectral intensities with a second polynomial order and filter length of 15. SERS spectra were also area normalized and mean cantered. Partial least squared discriminant analysis (PLS-DA) was used to build all models^20,21^. Each model had 3–7 principal components. True positive rate (TPR) of the model performance is reported for each model in Tables 1, 2, 3, 4, 5, 6, 7, 8, 9.

## Supplementary Information

Supplementary Figures.

## Data Availability

The datasets used and/or analyzed during the current study available from the corresponding author on reasonable request.

## References

[1] Oien, C. T. Forensic hair comparison: Background information for interpretation. *For. Sci. Commun.* **11** (2009).

[2] Wilson MR (1995). Extraction, PCR amplification and sequencing of mitochondrial DNA from human hair shafts. Biotechniques.

[3] Siegel JA, Mirakovits K, Siegel JA, Mirakovits K (2021). Hair. Forensic Science: The Basics.

[4] in *Global Hair Color Market: Industry Analysis and Forecast (2021–2027) by Usage, Application, End User, and Region*. Report No. 68529, (Maximize Market Research, 2020).

[5] Andrisano V, Gotti R, Di Pietra AM, Cavrini V (1994). HPLC analysis of oxidation hair dyes in permanent hair colorants. J. Liquid Chromatogr..

[6] Tanada N, Kashimura S, Kageura M, Hara K (1999). Practical GC/MS analysis of oxidation dye components in hair fiber as a forensic investigative procedure. J. Forens. Sci..

[7] Amorim A, Fernandes T, Taveira N (2019). Mitochondrial DNA in human identification: A review. PeerJ.

[8] Linch CA, Whiting DA, Holland MM (2001). Human hair histogenesis for the mitochondrial DNA forensic scientist. J. Forens. Sci..

[9] Barrett JA, Siegel JA, Goodpaster JV (2010). Forensic discrimination of dyed hair color: I UV-visible microspectrophotometry. J. Forens. Sci..

[10] Boll MS, Doty KC, Wickenheiser R, Lednev IK (2017). Differentiation of hair using ATR FT-IR spectroscopy: A statistical classification of dyed and non-dyed hairs. Forens. Chem..

[11] Kurouski D, Van Duyne RP (2015). In situ detection and identification of hair dyes using surface-enhanced Raman spectroscopy (SERS). Anal. Chem..

[12] Esparza I, Wang R, Kurouski D (2019). Surface-enhanced Raman analysis of underlaying colorants on redyed hair. Anal. Chem..

[13] Sharma B (2013). High-performance SERS substrates: Advances and challenges. MRS Bull..

[14] Wustholz KL (2010). Structure−activity relationships in gold nanoparticle dimers and trimers for surface-enhanced Raman spectroscopy. J. Am. Chem. Soc..

[15] Wei WY, White IM (2013). Inkjet-printed paper-based SERS dipsticks and swabs for trace chemical detection. Analyst.

[16] Virkler K, Lednev IK (2010). Raman spectroscopic signature of blood and its potential application to forensic body fluid identification. Anal. Bioanal. Chem..

[17] Riskin M, Tel-Vered R, Lioubashevski O, Willner I (2009). Ultrasensitive surface plasmon resonance detection of trinitrotoluene by a bis-aniline-cross-linked Au nanoparticles composite. J. Am. Chem. Soc..

[18] Sylvia JM, Janni JA, Klein J, Spencer KM (2000). Surface-enhanced Raman detection of 2, 4-dinitrotoluene impurity vapor as a marker to locate landmines. Anal. Chem..

[19] Higgins S, Kurouski D (2023). Surface-enhanced Raman spectroscopy enables highly accurate identification of different brands, types and colors of hair dyes. Talanta.

[20] Farber C (2020). Raman spectroscopy enables non-invasive identification of peanut genotypes and value-added traits. Sci. Rep..

[21] Gupta Y, Singla G, Singla R (2015). Insulin-derived amyloidosis. *Indian J*. Endocrinol. Metab..

