# Supplementary material for: Surface-enhanced Raman spectroscopy is capable of precise differentiation between re-dyed hair samples
Source: Sci Rep. 2023 May 1;13:7063. doi: 10.1038/s41598-023-34398-z (PMC10151316; doi:10.1038/s41598-023-34398-z)
Supplement: Supplementary file 1 — Supplementary Figures. [file 41598_2023_34398_MOESM1_ESM.docx]

**Surface-Enhanced Raman Spectroscopy is Capable of Precise Differentiation Between Re-Dyed Hair Samples**

Samantha Higgins^1^ and Dmitry Kurouski^1*^

^1^ Department of Biochemistry and Biophysics, Texas A&M University, College Station, TX 77843, USA.

*E-mail: [dkurouski@tamu.edu](mailto:dkurouski@tamu.edu) Tel: 979-458-3778

ORCID

Dmitry Kurouski: 0000-0002-6040-4213

Supporting Information

*
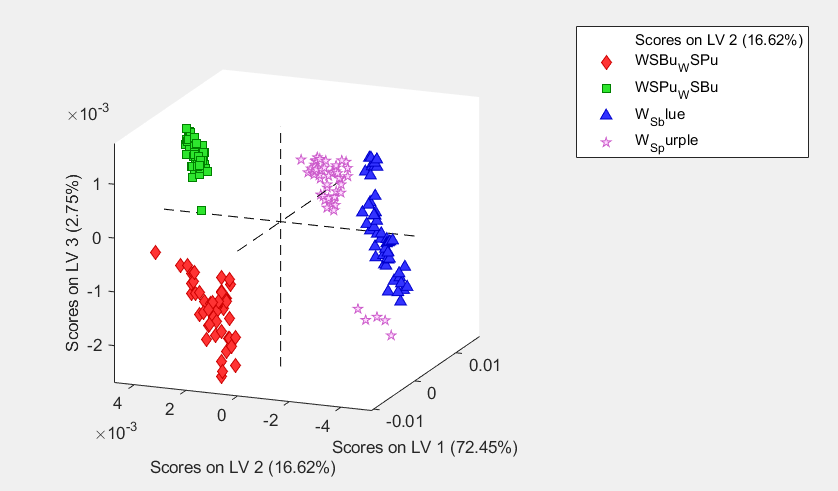
*

**Supporting Figure 1.** LV plot of colored hair layered with varying colors of hair dye after baselining, 1^st^ derivative, area normalizing, and mean centering. Red diamond cluster (Wella semi-permanent blue under Wella semi-permanent purple), green square cluster (Wella semi-permanent purple under Wella semi-permanent blue), blue triangle cluster (Wella semi-permanent blue), pink star cluster (Wella semi-permanent purple).

*
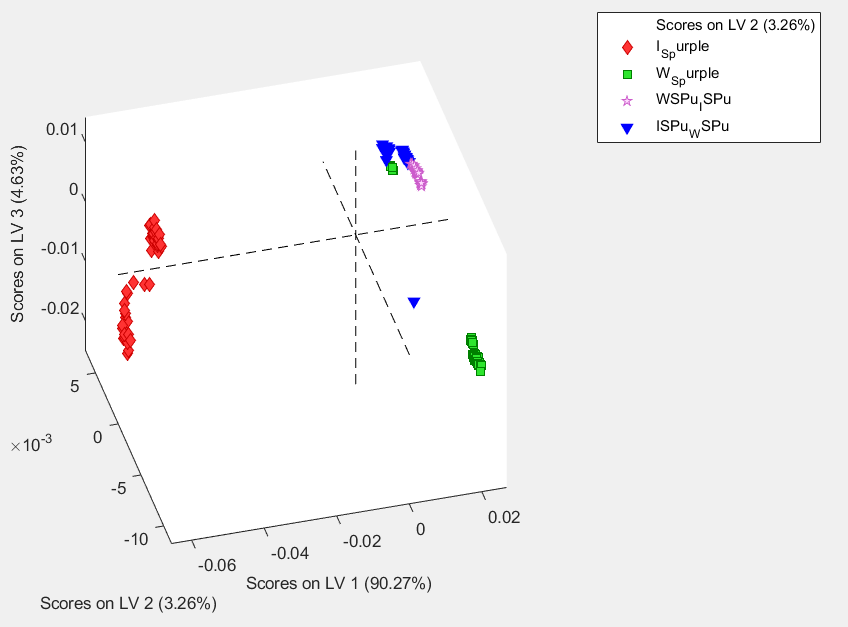
*

**Supporting Figure 2.** LV plot of colored hair layered with assorted brands of hair dye. These spectra were preprocessed with baselining, 1^st^ derivative, area normalizing, and mean centering before clustering. Red diamonds (Ion semi-permanent purple colorant), green squares (Wella semi-permanent purple colorant), light blue triangles (Ion semi-permanent purple dye under Wella semi-permanent purple dye), pink stars (Wella semi-permanent purple dye under Ion semi-permanent purple dye).

*
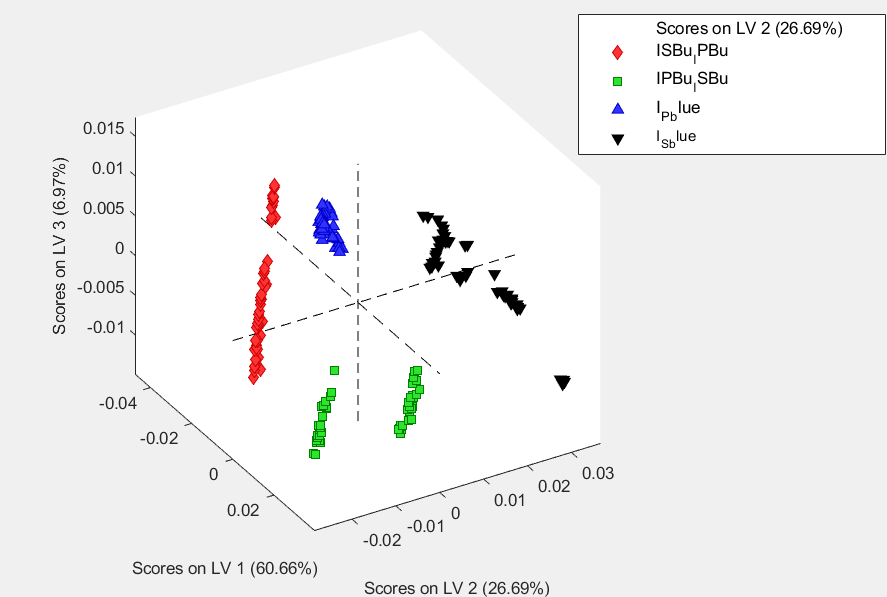
*

**Supporting Figure 3.** LV plot of colored hair layered with distinct types of hair dye baselined, 1^st^ derivative, area normalized, and mean centered SERS spectra. Red diamonds (Ion semi-permanent blue dye under Ion permanent blue dye), blue triangles (Ion permanent blue hair dye), black triangles (Ion semi-permanent blue), green squares (Ion permanent blue under Ion semi-permanent blue hair dye).

**
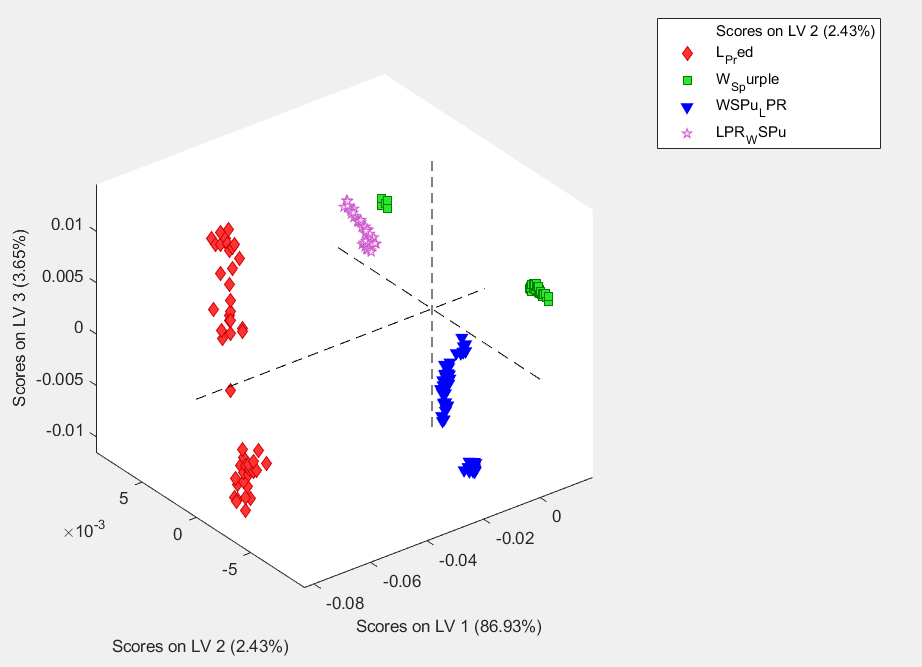
**

**Supporting Figure 4.** LV plot of colored hair layered with different type, brand, and color of hair dye baselined, 1^st^ derivative, area normalized, and mean centered SERS spectra. Red diamonds (L’Oréal permanent red), light blue triangles (Wella semi-permanent purple hair dye under L’Oréal permanent red hair dye), green squares (Wella semi-permanent purple), pink stars (L’Oréal permanent red under Wella semi-permanent purple).
